# Sticking Together Helps Cells Capture More Food and Keep It from the Cheaters

**DOI:** 10.1371/journal.pbio.1001123

**Published:** 2011-08-09

**Authors:** Richard Robinson

**Affiliations:** Freelance Science Writer, Sherborn, Massachusetts, United States of America

**Figure pbio-1001123-g001:**
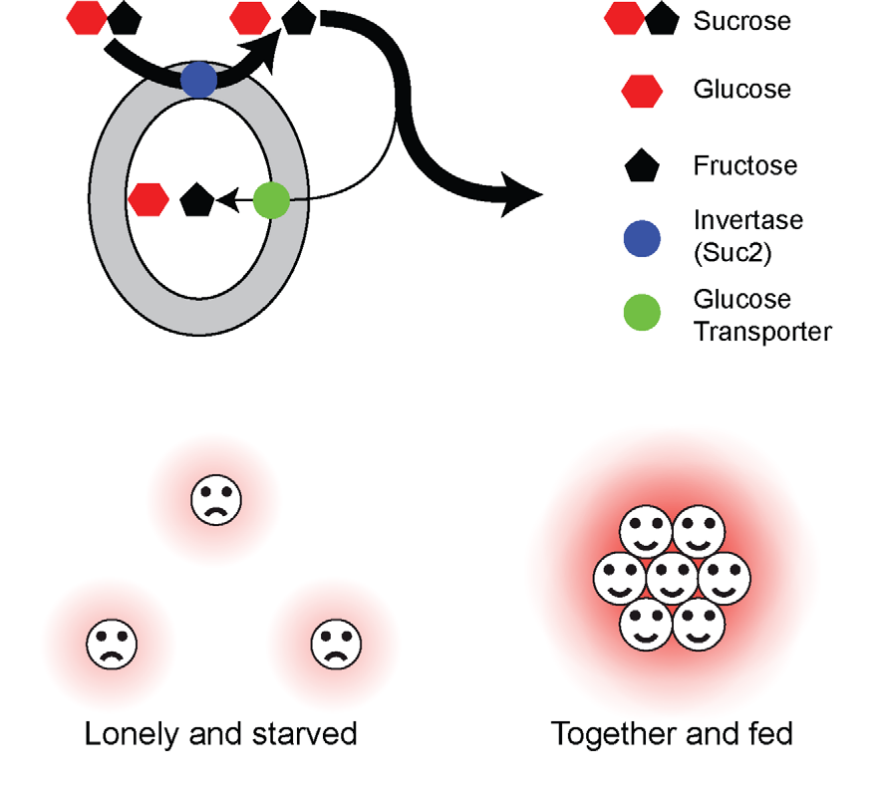
Multicellularity improves use of public goods. Invertase in the yeast cell wall releases glucose from sucrose (top). Individual cells take up too little glucose to grow (bottom left). Clumped cells harvest glucose released by their neighbors (bottom right).

[Fig pbio-1001123-g001]A multicellular organism is a kind of social group, and, as for all social groups, a key question is, what drives essentially selfish creatures to band together? The arms race of predation appears to be one answer—larger organisms make both better predators and worse prey. Another proposed answer is that multicellular creatures are better at concentrating and utilizing scarce nutrients than the same number of cells scattered about. But how can that proposition be tested? In this issue of *PLoS Biology*, John Koschwanez, Kevin Foster, and Andrew Murray use yeast cells, which can exist both independently and as aggregates, to show that under certain environmental conditions multicellular groups can grow when single cells cannot. These groups' ability to husband their resources not only allows them to flourish in trying conditions, but appears to protect them against the slackers in their midst as well.

Yeasts need sugar to grow, but they cannot absorb common table sugar, sucrose. To take advantage of the relatively complex sucrose molecule, they secrete the enzyme invertase, which is retained in the cell wall, where it hydrolyzes sucrose into its constituents, glucose and fructose. Those monosaccharides can then be absorbed by the yeast cell. But the absorption isn't efficient—most of the simple sugars diffuse away before they can be captured by the yeast cell that hydrolyzed them. Those dispersed monosaccharides can then be used by other yeast cells, including ones that don't make their own invertase—cheaters, so to speak, that benefit from the labor of others without contributing anything to the group themselves.

The authors used a combination of modeling and experiments to ask whether multicellularity did indeed offer yeasts an advantage when food was scarce. The model simulated the secretion of invertase, its retention in the cell wall, its hydrolysis of sucrose, and the diffusion and capture of monosaccharides. The authors compared two situations: 30 single cells, uniformly dispersed through the medium, and single clump of 30 cells. They found that clumped cells, which are much closer to one another than single cells, have access to almost ten times as much monosaccharide as the single cells, predicting that clumps of cells could grow in low sucrose concentration but that dispersed populations of cells could not. The benefit of clumping in this model reached a maximum at about 1,000 cells, after which diffusion was too slow to adequately supply cells in the center of the clump.

The authors tested their prediction by comparing the growth of equal numbers of clumped and dispersed cells. At low sucrose concentrations, clumps could grow but dispersed cells could not. The beneficial effect of clumping was specifically dependent on both invertase production and secretion; when either function was lost, clumped cells did no better than single cells. This confirmed that multicellular groups can forage for nutrients better than single cells.

But when cells that could not make invertase—the cheaters—were grown with cells that could, the authors discovered an interesting phenomenon. When cells were widely dispersed, there was no disadvantage to being a cheater, because most of the monosaccharides escape the cells that liberated them. But because each cell in a clump is close to a neighbor, the clumped cells that make invertase have privileged access to the monosaccharides that they liberate, leaving less for the cheaters. This difference may help to explain why cheating is rare even though there is a “fitness cost” to making invertase, which the authors showed amounts to about one-third of one percent; not huge, but not trivial either, and potentially enough to benefit a cheater amidst a sea of single-celled invertase producers.

The authors suggest that clumping, and the consequent nutritional benefit to all the cells in the aggregate, is one plausible mechanism for the origin of multicellularity. Since secretion of enzymes is a common strategy among many types of prokaryotes and unicellular eukaryotes, it may have contributed to development of multicellularity in multiple lineages. According to this model, the cell specialization that characterizes most modern eukaryotes would have developed after the initial sticking together of a group of homogenous cells.

**Koschwanez J, Foster K, Murray A (2011) Sucrose Utilization in Budding Yeast as a Model for the Origin of Undifferentiated Multicellularity. doi:10.1371/journal.pbio.1001122**

